# Discovery of SARS-CoV-2 M^pro^ peptide inhibitors from modelling substrate and ligand binding†

**DOI:** 10.1039/d1sc03628a

**Published:** 2021-09-06

**Authors:** H. T. Henry Chan, Marc A. Moesser, Rebecca K. Walters, Tika R. Malla, Rebecca M. Twidale, Tobias John, Helen M. Deeks, Tristan Johnston-Wood, Victor Mikhailov, Richard B. Sessions, William Dawson, Eidarus Salah, Petra Lukacik, Claire Strain-Damerell, C. David Owen, Takahito Nakajima, Katarzyna Świderek, Alessio Lodola, Vicent Moliner, David R. Glowacki, James Spencer, Martin A. Walsh, Christopher J. Schofield, Luigi Genovese, Deborah K. Shoemark, Adrian J. Mulholland, Fernanda Duarte, Garrett M. Morris

**Affiliations:** Chemistry Research Laboratory, Department of Chemistry and the Ineos Oxford Institute for Antimicrobial Research 12 Mansfield Road Oxford OX1 3TA UK fernanda.duartegonzalez@chem.ox.ac.uk christopher.schofield@chem.ox.ac.uk; Department of Statistics, University of Oxford 24-29 St Giles' Oxford OX1 3LB UK garrett.morris@stats.ox.ac.uk; Centre for Computational Chemistry, School of Chemistry, University of Bristol Cantock's Close Bristol BS8 1TS UK adrian.mulholland@bristol.ac.uk; Intangible Realities Laboratory, School of Chemistry, University of Bristol Cantock's Close Bristol BS8 1TS UK; School of Biochemistry, University of Bristol, Medical Sciences Building University Walk Bristol BS8 1TD UK deb.shoemark@bristol.ac.uk; RIKEN Center for Computational Science 7-1-26 Minatojima-minami-machi, Chuo-ku Kobe Hyogo 650-0047 Japan; Diamond Light Source Ltd, Harwell Science and Innovation Campus Didcot OX11 0DE UK; Research Complex at Harwell, Harwell Science and Innovation Campus Didcot OX11 0FA UK; Biocomp Group, Institute of Advanced Materials (INAM), Universitat Jaume I 12071 Castello Spain; Food and Drug Department, University of Parma Parco Area delle Scienze, 27/A 43124 Parma Italy; Univ. Grenoble Alpes, CEA, IRIG-MEM-L_Sim 38000 Grenoble France luigi.genovese@cea.fr

## Abstract

The main protease (M^pro^) of SARS-CoV-2 is central to viral maturation and is a promising drug target, but little is known about structural aspects of how it binds to its 11 natural cleavage sites. We used biophysical and crystallographic data and an array of biomolecular simulation techniques, including automated docking, molecular dynamics (MD) and interactive MD in virtual reality, QM/MM, and linear-scaling DFT, to investigate the molecular features underlying recognition of the natural M^pro^ substrates. We extensively analysed the subsite interactions of modelled 11-residue cleavage site peptides, crystallographic ligands, and docked COVID Moonshot-designed covalent inhibitors. Our modelling studies reveal remarkable consistency in the hydrogen bonding patterns of the natural M^pro^ substrates, particularly on the N-terminal side of the scissile bond. They highlight the critical role of interactions beyond the immediate active site in recognition and catalysis, in particular plasticity at the S2 site. Building on our initial M^pro^-substrate models, we used predictive saturation variation scanning (PreSaVS) to design peptides with improved affinity. Non-denaturing mass spectrometry and other biophysical analyses confirm these new and effective ‘peptibitors’ inhibit M^pro^ competitively. Our combined results provide new insights and highlight opportunities for the development of M^pro^ inhibitors as anti-COVID-19 drugs.

## Abstract 2

The main protease (M^pro^) of SARS-CoV-2 is central to viral maturation and is a promising drug target. *In silico* methods reveal structural aspects of how it binds to its 11 natural cleavage sites, the design of novel peptide inhibitors, and insights into drug design.

## Graphical abstract

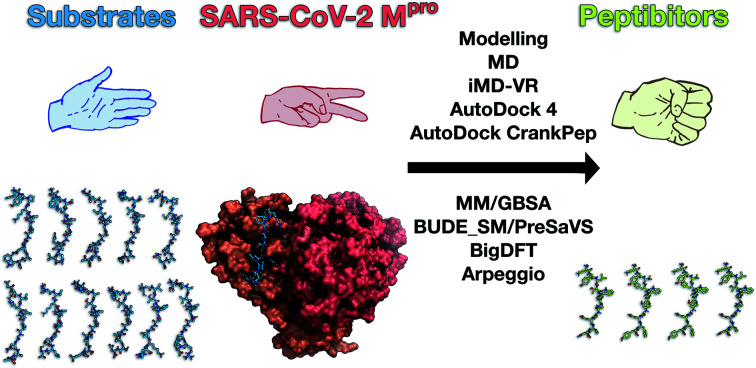

## 1. Introduction

Severe acute respiratory syndrome coronavirus 2 (SARS-CoV-2) is the etiological agent of coronavirus disease 2019 (COVID-19) that caused the World Health Organization to declare a global pandemic in March 2020. At the time of writing, >210 million COVID-19 cases and >4.4 million deaths have been reported worldwide.^1^ A key step in maturation of SARS-CoV-2, a single-stranded positive-sense RNA virus, is hydrolysis of its polyproteins pp1a and pp1ab. Most of the cleavage events—at 11 sites—are performed by the SARS-CoV-2 main protease (M^pro^; 3 chymotrypsin-like or 3CL proteinase, 3C-like protease, 3CL^pro^; or non-structural protein 5, Nsp5).

M^pro^ is a nucleophilic cysteine protease, which in solution is predominantly homodimeric. Each protomer consists of three domains, and the active site contains a cysteine–histidine catalytic dyad, Cys-145 and His-41, located near the dimer interface.^2^ SARS-CoV-2 M^pro^ is 96% identical to the M^pro^ of SARS-CoV, which causes SARS.^3^ Dimerisation of M^pro^ is proposed as a prerequisite for catalysis: the N-terminus of one protomer contributes part of the active site of the other.^4^ Indeed, the monomeric form of SARS-CoV M^pro^ is reported to be inactive.^5^ Evidence from non-denaturing mass spectrometry (MS)-based assays indicates that M^pro^ monomers are not only inactive (at least with tested substrates), but do not bind 11-mer substrates with high affinity.^6^

**Fig. 1:**
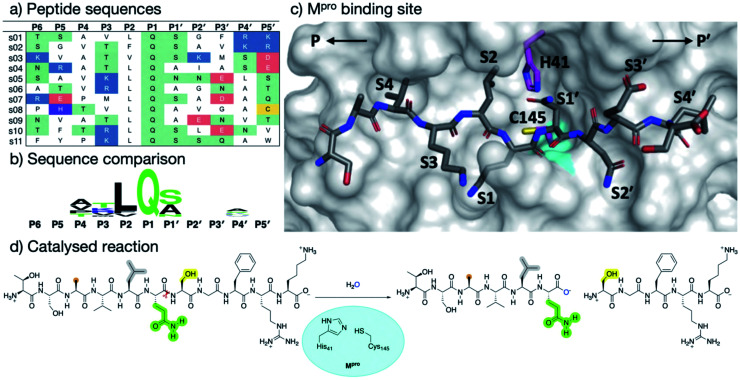
Substrates processed by SARS-CoV-2 M^pro^. (a) The 11 SARS-CoV-2 M^pro^ cleavage sites, and the corresponding 11-residue peptides, s01–s11; positively/negatively charged residues are blue/red, respectively; histidine is purple; residues with polar sidechains are green; and cysteine is yellow. (b) Comparison between the 11 substrate sequences (generated by WebLogo)^9^ highlighting the completely conserved Gln at P1 and the highly conserved Leu at P2. (c) View of an energy minimised model, built using *apo* M^pro^ (PDB: 6yb7, light grey surface),^10^ of M^pro^ complexed with s05 (dark grey sticks); subsites S4–S4′ are labelled. The oxyanion hole formed by the M^pro^ backbone NHs of Gly-143, Ser-144 and Cys-145 is cyan. (d) The reaction catalysed by M^pro^ exemplified by s01. Substrate residues important in recognition (see main text) are highlighted.

SARS-CoV-2 M^pro^ and SARS-CoV M^pro^ have similar substrate specificities, both recognizing the motif: [P4:Small] [P3:X] [P2:Leu/Phe/Val/Met] [P1:Gln]↓[P1′:Gly/Ala/Ser/Asn], “Small” denoting Ala, Val, Pro or Thr; “X” any residue; and “↓” the scissile amide (Fig. 1).^7,8^ In part, because such sequences are not known to be recognised by any human protease, M^pro^ represents an attractive drug target.^4^ Although no clinically approved M^pro^ drugs are available, small molecule inhibitors and peptidomimetics have been designed to inhibit SARS-CoV M^pro^ and, more recently, SARS-CoV-2 M^pro^.^11,12^ Indeed, a covalent M^pro^ inhibitor from Pfizer has recently entered clinical trials.^13,14^

Multiple crystallographic and computational modelling studies concerning the M^pro^ mechanism^15–18^ and inhibition are available;^19–24^ the CORD-19 database^25^ documents many such studies. It is proposed that during M^pro^ catalysis, His-41 deprotonates the Cys-145 thiol, which reacts with the carbonyl of the scissile amide to give an acyl-enzyme intermediate. This intermediate is stabilised by a hydrogen bond network that holds the scissile amide carbonyl in an ‘oxyanion hole’. The C-terminal part of the product likely leaves the active site at this stage. The acyl-enzyme intermediate is subsequently hydrolysed with loss of the N-terminal product regenerating active M^pro^. Computational and mechanistic studies on SARS-CoV M^pro^^26–29^ and SARS-CoV-2 M^pro^^30–32^ suggest that in the resting state His-41 and Cys-145 are likely neutral and that the protonation states of nearby histidines (*e.g.* His-163, 164, and 172) affect the structure of the catalytic machinery—although it has been suggested in SARS-CoV M^pro^ that the protonation state of the catalytic dyad may change in the presence of an inhibitor or substrate.^33^ A different picture has been obtained from neutron crystallographic studies, which indicate that an ion pair form of the dyad is favoured at pH 6.6.^34^ While neutron crystallography, in principle, enables the direct determination of hydrogen atom positions, questions remain about how pH and the presence of active site-bound ligands influence the precise—and likely dynamic—protonation state(s) of the dyad.

Important questions remain regarding M^pro^ catalysis, including to what extent the active site protonation state, solvent accessibility, induced fit, and substrate sequence influence activity. The lack of this knowledge makes it difficult to carry out effective computational studies on M^pro^ catalysis and inhibition.

With the aim of helping to combat COVID-19, in April 2020 we embarked on a collaborative effort involving weekly virtual meetings, initially to investigate the relationship between M^pro^ substrate selectivity and activity. We employed an array of classical molecular mechanics (MM) and quantum mechanical (QM) techniques, including non-covalent and covalent automated docking, molecular dynamics (MD) simulations, density functional theory (DFT), combined quantum mechanics/molecular mechanics (QM/MM) modelling, and interactive MD in virtual reality (iMD-VR). Our results provide consensus atomic-level insights into the interactions of M^pro^ with 11-residue peptides derived from the 11 natural cleavage sites (named “s01” to “s11”, in order of occurrence in the viral polyprotein, Fig. 1a). The identification of key interactions between M^pro^ and its substrates, together with analysis of fragment/inhibitor structures,^35,36^ led to the design of peptides proposed to bind more tightly than the natural substrates, several of which inhibit M^pro^. The results are freely available *via* GitHub (https://github.com/gmm/SARS-CoV-2-Modelling).

## 2. Results and discussion: understanding substrate binding and recognition

### 2.1 Protonation state of the catalytic dyad

The protonation state of the dyad following substrate binding was studied with s01-bound (Fig. 1a) M^pro^^37^ using QM/MM umbrella sampling simulations at the DFTB3/MM and ωB97X-D/6-31G(d)/MM levels of theory (Section S1.1†). The protonation states of nearby histidines were also evaluated (Table S2.1 and Fig. S2.5, S2.6†).

**Fig. 2:**
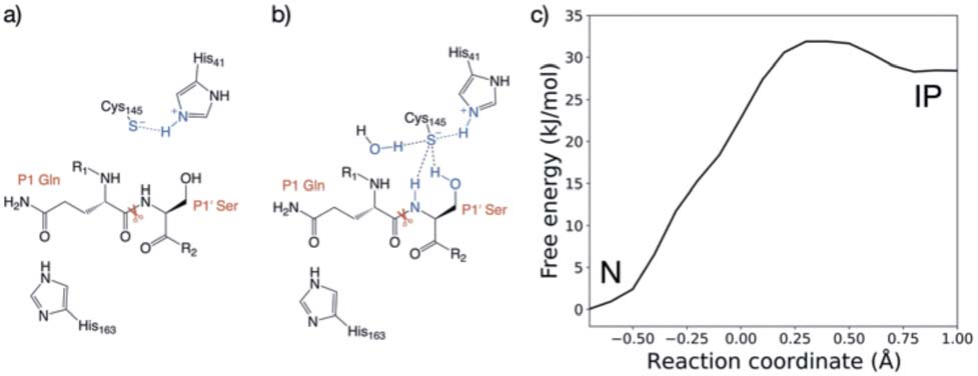
QM/MM umbrella sampling of the proton transfer in the catalytic dyad. Representation of the interactions of the Cys-145 thiolate in the ion pair (IP) state from the (a) forwards and (b) backwards simulations. (c) Free energy profile for interconversion of the neutral (N) and ion pair states of the dyad in the HIE-163 system, from the combined forwards and backwards QM/MM umbrella sampling MD simulations, corrected to the ωB97X-D/6-31G(d)/MM level of theory.

His-41 was treated as Nδ-protonated in the neutral state of the dyad, as reported by Pavlova *et al.* to be preferred for both uncomplexed and N3 inhibitor-bound M^pro^ based on MD studies.^30^ The protonation state of the dyad-neighbouring His-163, which interacts with Tyr-161, Phe-140 and the substrate P1 Gln sidechain, was also studied. Three His-163 protonation states were considered: (i) Nδ-protonated, neutral (“HID”); (ii) Nε-protonated, neutral (“HIE”); and (iii) Nδ and Nε-protonated, positively charged (“HIP”).^38^ For all three His-163 protonation states, the forward trajectories (neutral, N to ion pair, IP) showed the anionic Cys-145 form to be stabilised solely by interaction with positively charged His-41 (Fig. 2a and S2.2A–C†), and the thiolate in an unreactive conformation for nucleophilic attack. This suggests that such a zwitterionic state is transient, with concerted proton transfer and simultaneous nucleophilic attack of the thiolate onto the scissile amide carbon being more likely than a stepwise mechanism.^15^ By contrast, the backwards PT trajectories (from IP to N) showed stabilisation of the Cys-145 thiolate by His-41 and the P1 Gln backbone N–H, in the case of HID-163 and HIP-163 (Fig. S2.2E, F†). For HIE-163, additional stabilisation comes from interactions with the backbone N–H and the hydroxyl of the P1′ Ser, and an additional water that diffuses into the active site (Fig. 2b and S2.2D†).

The zwitterionic state with HID-163 was less stable than the neutral state and the zwitterionic states with HIE-163 and HIP-163 (Fig. S2.1C†). This was due to perturbation of the interaction network with Tyr-161 and Phe-140, suggesting that a Nδ-protonated His-163 is unlikely. Double protonation of His-163 results in a loss of both interactions in the forwards and backwards PT trajectories. Despite both HIP-163 and HIE-163 giving similar PT free energy profiles, the loss of these interactions suggests HIP-163 is unfavourable for productive catalysis. These QM/MM results therefore suggest that an Nε-protonated neutral His-163 is most likely. Along with conserving interactions with Tyr-161 and Phe-140, an Nε-protonated His-163 also formed a hydrogen bond with the P1 Gln side chain (Fig. 2a and b), an interaction not observed in PT trajectories with HID-163 and HIP-163.

Considering that DFTB3 overestimates the proton affinity of methylimidazole, it is expected that this method will over-stabilise the zwitterionic state relative to the neutral state.^39^ To account for this, the backwards PT reaction with a Nδ-protonated His-163 was modelled at the ωB97X-D/6-31G(d)/MM level of theory. This showed the zwitterionic state was 24.3 kJ mol^−1^ above the neutral state, an increase of 26.4 kJ mol^−1^ compared to DFTB3/MM (Fig. S2.3†). Applying the free energy difference between ωB97X-D/6-31G(d) and DFTB3 at each reaction coordinate value as a correction to the combined QM/MM free energy profile in the case of HIE-163, the neutral catalytic dyad is preferred, with the ion pair being 28.5 kJ mol^−1^ higher in energy than the neutral state (Fig. 2c). Similar results were obtained with a different QM approach (Fig. S2.4 and associated ESI Movie†).

### 2.2 Models of SARS-CoV-2 M^pro^–substrate peptide complexes

To understand substrate specificity and to assess their relative binding affinities, we constructed 11 models of SARS-CoV-2 M^pro^ complexed with its cleavage site-derived substrates^40^ as 11-amino acid peptides, from P6 to P5′ (Section S1.2; Fig. 1a and S2.7†). We refer to these peptides as ‘substrates’ as their hydrolytic sites are all cleaved by M^pro^ (*vide infra*). The substrates were modelled in crystallographic chain A of the M^pro^ dimer (PDB: 6yb7)^10^ with a neutral dyad; unless otherwise stated, all M^pro^ residue numbers and names in the following discussions refer to chain A. Initial models were subjected to three independent explicit-solvent MD simulations each of 200 ns.

Three of the 11 cleavage site-derived peptides (s01, s02, and s05) were also modelled with interactive MD using virtual reality (iMD-VR), as an alternative to comparative modelling and traditional MD. iMD-VR provides an immersive 3D environment for users to interact with physically rigorous MD simulations.^41–43^ The three substrates were chosen because s01 and s02 have the highest relative efficiencies (of SARS-CoV M^pro^) of all substrates; while s05 has the second-lowest catalytic efficiency but the same P2 and P1 residues as s01.^44^

Throughout both the explicit-solvent MD and implicit-solvent iMD-VR simulations, all the substrates remained tightly bound in the active site (Fig. S2.8–S2.11†). Substrate backbone stability was maintained especially in the central region, with only the N- and C-terminal residues showing substantial flexibility. Local sidechain fluctuations were present, notably at the solvent-facing P3 residue (Fig. 1c and S2.9†). C-terminal P′-residues consistently fluctuated more than the N-terminal P-side residues, likely in part because of fewer protein–substrate hydrogen bonds on the P′ side (*vide infra*).

#### 2.2.1 Conserved hydrogen bond interactions

**Fig. 3:**
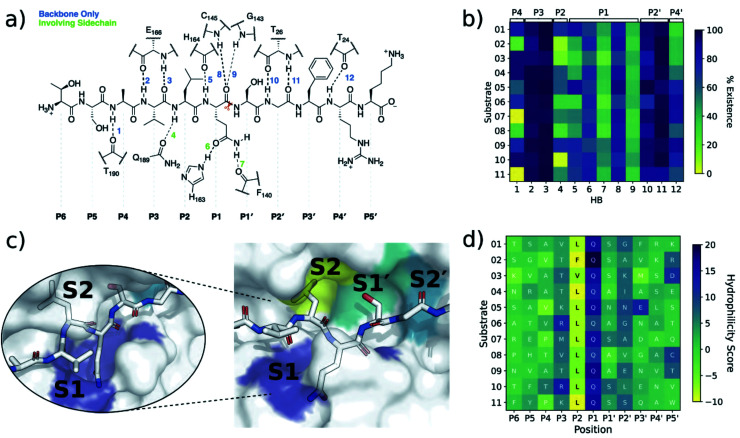
Interactions between SARS-CoV-2 M^pro^ and its substrates. (a) M^pro^–substrate hydrogen bonds (HBs) exemplified by substrate s01. (b) An annotated heat-map showing the frequency of each HB, with blue indicating highest frequency. Frames were extracted every ns from 600 ns of cumulative explicit-solvent MD conducted per system. (c) Close-up of the MD-generated binding mode of SARS-CoV-2 M^pro^-substrate s01 with subsites S1, S2, S1′ and S2′ labelled. Different views of the S1 subsite are shown, emphasizing the deep S1 pocket that accommodates the P1 Gln sidechain. Subsite surface colour corresponds to the hydrophilicity score, with hydrophobic subsites shown in yellow, hydrophilic subsites in dark blue, and amphiphilic subsites in turquoise. (d) Hydrophilicity map for the 11 substrates calculated as the sum of hydrophilic interactions (dark blue) subtracted from the sum of hydrophobic interactions (yellow). Interactions were identified from substrate:M^pro^ frames using Arpeggio^45^ (Methods Section S1.5†).

Crystallographic studies on SARS-CoV M^pro^ revealed the importance of hydrogen bonds in binding of substrate s01,^37^ which has the same cleavage site sequence in SARS-CoV-2 (Fig. 1a). To investigate whether this was true for SARS-CoV-2 M^pro^ complexed with its 11 substrates, we analysed the hydrogen bond (HB) prevalence at each subsite in both explicit-solvent MD and implicit-solvent iMD-VR simulations. Twelve HBs were consistently identified (Fig. 3) and their distance and angular distributions analysed (Fig. S2.12†).

In both explicit-solvent MD and iMD-VR simulations, all 11 substrates are primarily held in place by four consistently formed backbone–backbone HBs: Glu-166 at S3 (2 & 3) and Thr-26 at S2′ (10 & 11; Fig. 3). Backbone HBs 1 and 12 further from the cleavage site show greater variation between the MD and iMD-VR studies. Although P1 Gln is conserved in all 11 M^pro^ cleavage sites (Fig. 1a and b), HBs 5–9 formed in the S1 site are observed less often than 2, 3, 10 and 11, but outnumber other sites. In both types of simulations, HB 8 from the Cys-145 backbone amide is consistently formed, suggesting that this could play a fundamental role in catalysis. HB 8 forms part of the oxyanion hole, which stabilises the tetrahedral intermediate formed upon nucleophilic attack by Cys-145 Sγ on the scissile amide. M^pro^'s exquisite specificity for Gln at P1 is likely due to formation of HB 6 with His-163, and to a lesser extent HB 7, along with the narrowness of the S1 pocket accommodating the Gln sidechain in an extended conformation.

#### 2.2.2 Hydrophobicity analysis

Hydrophilicity analysis shows that the S1 subsite is substantially hydrophilic in all 11 substrate–M^pro^ complexes, while S2 is consistently hydrophobic (Fig. 3d). In accord with the MD-HB analysis (Fig. 3b), the conservation of subsite interactions decreases further from the cleavage site. Although S3 and S2′ show a slight bias towards hydrophilic interactions, none of the other subsites show a consistent pattern.

#### 2.2.3 Other non-covalent interactions

**Fig. 4:**
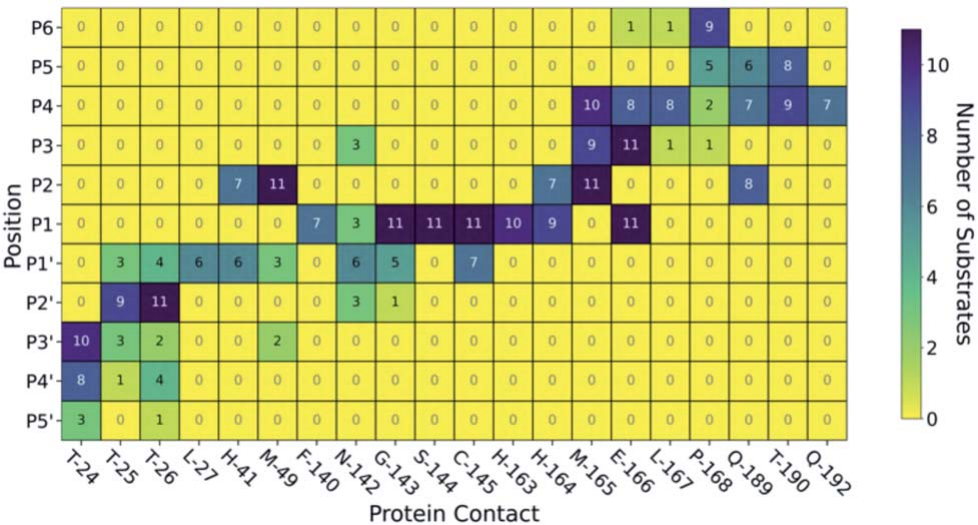
M^pro^–substrate contacts. Map of HBs and other non-covalent interactions between the 11 substrates and M^pro^ from Arpeggio analysis of the most representative pose for each substrate generated by MD. Dark blue indicates the interaction is formed by all 11 substrates at that substrate position, while yellow indicates no substrates form this interaction.

Beyond HBs, several other interaction types are conserved across the substrates; these were identified by running Arpeggio^45^ on snapshots extracted from the explicit-solvent MD simulations (Fig. 4). Six of the eight most common P1 interactions are present in most (*i.e.*, ≥9/11) substrates. This includes the previously described HBs between the P1 backbone oxygen and the backbone NHs of Cys-145 (HB 8) and Gly-143 (HB 9) that constitute the oxyanion hole, as well as HB 6 (His-163) and HB 7 (Phe-140) that stabilise the P1 Gln sidechain. In addition, interactions with Ser-144, His-163, His-164, Glu-166 and, to a lesser extent, Phe-140 were prevalent at P1. None of the other subsites show this level of consistency in residue-level contacts, although some interactions such as hydrophobic contacts with Met-49 and Met-165 were always present at P2. Furthermore, important stabilising backbone HBs (HBs 2–3 between Glu-166 and P3; and HBs 10–11 between Thr-26 and P2′) were conserved in all substrates. Finally, P′ interactions are less common than those on the P side (Fig. 4). The same trend was found when docking s01, s02, and s05 using iMD-VR, where P′ residues tended to be more flexible than P-side residues.

We further analysed the energetic contributions of each M^pro^ residue using the Molecular Mechanics-Generalised Born Surface Area (MM-GBSA) method (Section S2.3.1†),^46–49^ which highlighted hotspot residues that were also recognised by Arpeggio as conserved contacts.

#### 2.2.4 Density functional theory analysis of the interaction network

**Fig. 5:**
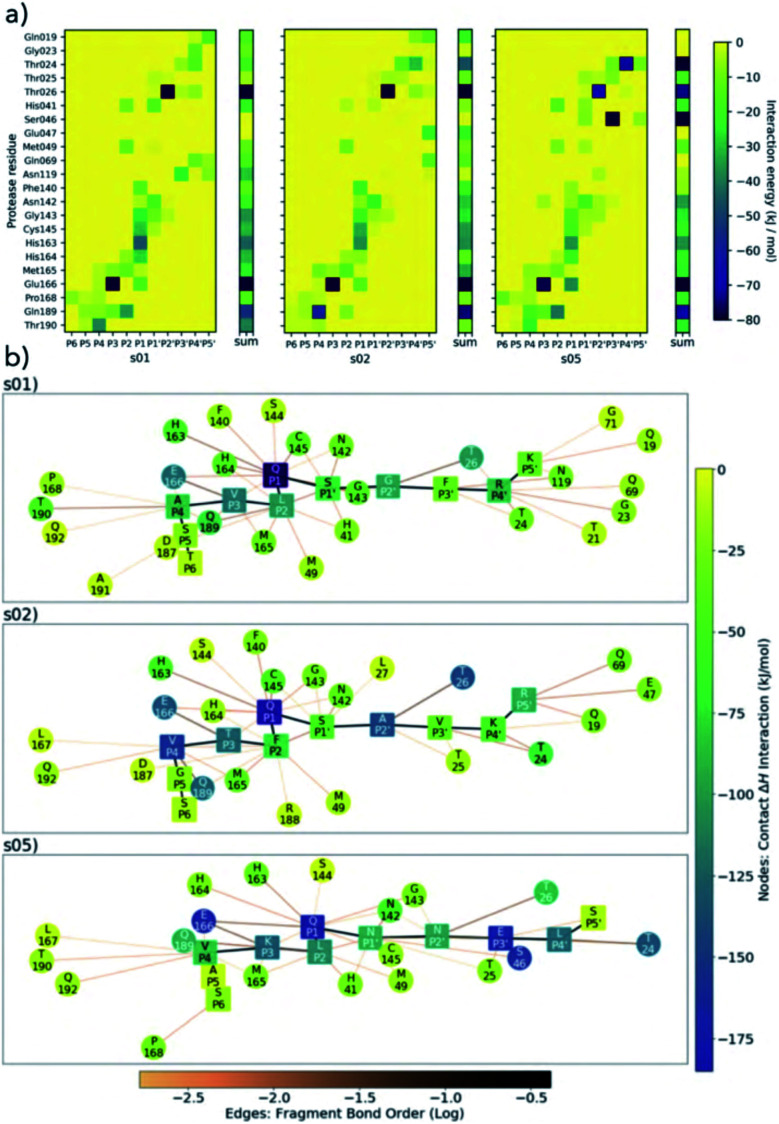
BigDFT analysis of M^pro^–substrate interactions. (a) Heatmaps showing QM interaction energies between 22 selected residues of M^pro^ and s01, s02 and s05. (b) QM interaction networks where node colour indicates interaction strength, from dark blue (strongest) through green to yellow (weakest). Square nodes denote substrate, while circular ones denote M^pro^. The thickness and colour of the edges show the fragment bond order between residues, a unitless measure associated with bond strength and analogous to bond order; black is strongest, orange is weakest.^51^ Interaction energies and bond orders were computed using BigDFT and ensemble-averaged results of MD snapshots.

We performed linear-scaling DFT (BigDFT^50^) calculations using representative snapshots extracted from explicitly solvated MD trajectories of M^pro^ complexed with s01, s02, and s05. By automatically decomposing large molecular systems into coarse-grained subsets of atoms (or ‘fragments’) in an unbiased manner,^51^ quantities like inter-fragment bond order and interaction strengths, *E*_cont_, can be easily calculated (Fig. 5a, S2.18–S2.21 and Section S2.4†). This analysis supports the essential roles of Glu-166 and Thr-26, with interactions observed in all three peptides s01, s02, and s05, consistent with the HB analysis described earlier (Fig. 3). Gln-189 consistently hydrogen-bonds with P2 (HB-4) in s01 and s05, but rarely in s02. This weakening of HB-4 in P2 may be due the greater bulk of Phe in s02 (Fig. 5b).

Conserved contacts are present in the three substrates between Cys-145 and both P1 and P1′ residues. Interactions between His-41 and P2/P1′ are observed for s01 and s05, and between Glu-166 and P1/P3 (and, to some extent, to P4) for all three substrates. This analysis singles out the character of s02, which is dominated by the bulky character of its P2 Phe. Substitutions at P2 may have a substantial effect on the interaction network close to the catalytic site. While the P side exhibits an interconnected character especially from P1 to P4 (Fig. 5b), the network on the P′ side has a more linear character, once again indicating that hotspot residues responsible for binding are present on the P side. Distributions of *E*_cont_ are shown in Fig. S2.20.†

The following trends emerge from our studies on M^pro^ in complex with models of its 11 substrates: (i) binding stability is partly conferred by a series of HBs from P4 to P4′, in particular between the backbones of M^pro^ Glu-166 and Thr-26 and substrate positions P3 and P2′ respectively, as well as HBs involving the conserved P1 Gln sidechain; (ii) substrate residues N-terminal of the cleavage site (P side) form more, and more consistent, contact interactions with M^pro^ compared to the P′ side, with interactions at Met-49, Gly-143, Ser-144, Cys-145, His-163, His-164, Met-165 and Glu-166 being most conserved. We conclude that the S1 and S2 pockets are prime targets for active site substrate-competing inhibitor design due to their well-defined hydrophilic character, large energy contributions to substrate binding, and vital conserved hydrogen bonds in S1 for substrate recognition.

#### 2.2.5 Conformational plasticity in M^pro^ crystal structures

**Fig. 6:**
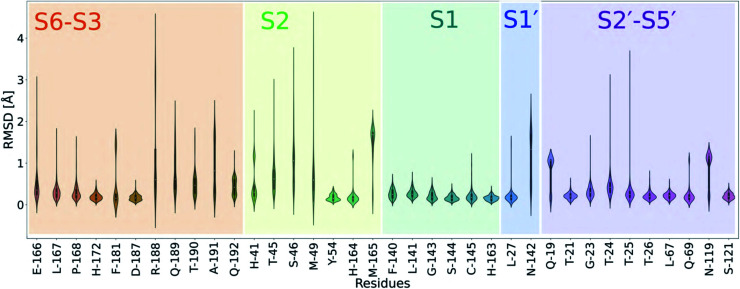
Analysis of the active site plasticity of 333 M^pro^ co-crystal structures. Active site residues (residues 19, 21, 23–27, 41, 45, 46, 49, 54, 67, 69, 119, 121, 140–145, 163–168, 172, 181, 187–192) were chosen based on the MD analysis of the 11 substrate–M^pro^ models and correspond to all M^pro^ residues that contact any substrate. The violin plots show the distributions of per-residue heavy atom RMSD values between the 333 M^pro^–ligand co-crystal structures^53^ and a reference uncomplexed structure (PDB 6yb7).^10^ Each M^pro^ subsite is colour-coded.

Previous studies have compared the dynamics of ligand binding sites across SARS-CoV-2, SARS-CoV and MERS-CoV M^pro^.^52^ Here, we investigated the conformational plasticity of the SARS-CoV-2 M^pro^ active site upon binding by comparing 333 M^pro^:ligand co-crystal structures obtained from Fragalysis^53^ to a reference *apo* structure of M^pro^, PDB entry 6yb7 (ref. 10) (Fig. 6). A high degree of plasticity was observed at residues Thr-24, Thr-25, His-41, Thr-45, Ser-46, Met-49, Asn-142, Met-165, Glu-166, Arg-188, Gln-189 and Ala-191. Unsurprisingly, the S1 subsite is particularly rigid, with almost no change in residue conformations across all 333 crystal structures.

In all cases, P1 Gln recognition is mainly driven by interactions with Gly-143, Ser-144, Cys-145 (oxyanion hole) and His-163 and Phe-140. The S2 subsite, however, is highly flexible, especially at Thr-45, Ser-46 and Met-49. Although P2 is conserved in terms of hydrophobicity (Leu, Phe, Val), the S2 pocket is highly flexible and can adapt to accommodate functional groups of varying sizes, including aliphatic and aromatic groups. The outer regions of the active site (S3–S6 and S2′–S5′) vary in flexibility, echoing our MD simulations.

### 2.3 Monitoring of substrate sequence hydrolysis by mass spectrometry

To rank the SARS-CoV-2 M^pro^ preferences for hydrolysis of the 11 cleavage sites, we monitored turnover of 11-mer peptides by solid-phase extraction (SPE) coupled to mass spectrometry (MS). Interestingly, after the N-terminal autocleavage site s01, s11 was the next preferred substrate for catalysis (Fig. S2.22†). Peptides s06, s02, and s10 were hydrolysed less efficiently than s11. Slow turnover was observed for s07 and s09. Evidence for low turnover of s05 was obtained after prolonged incubation with M^pro^ (9.56%) (Fig. S2.23†). Under our standard conditions, no evidence for cleavage was observed for s03, s04, and s08.

**Fig. 7:**
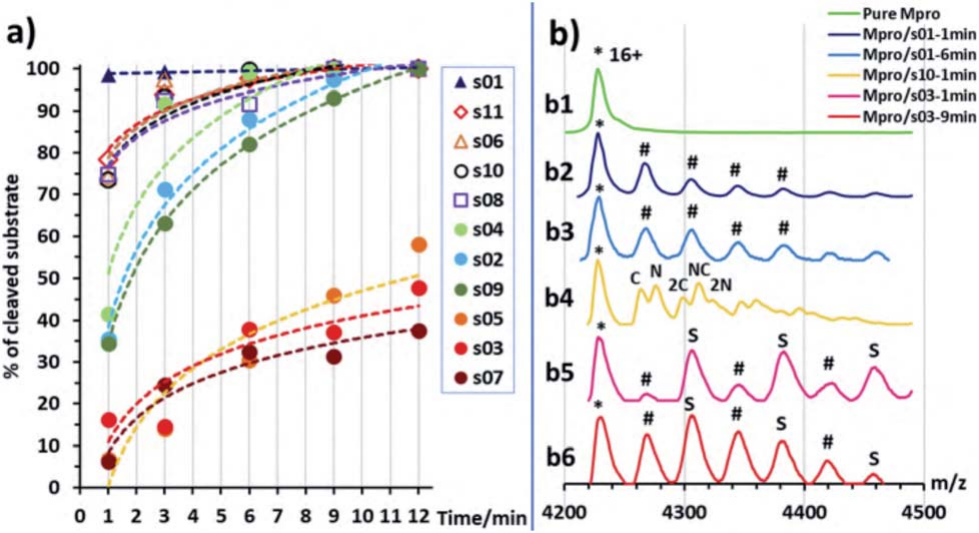
Non-denaturing mass spectrometry of M^pro^ substrate turnover. (a) Substrate turnover *versus* incubation time as measured by non-denaturing MS. Trend lines are given for visual guidance only. (b) Examples of mass spectra showing normalized intensity in the *m*/*z* region around the 16+ charge state of M^pro^ dimer (asterisk, *): (b1) pure M^pro^ solution (5 μM); (b2) M^pro^ and s01 solution after 1 min incubation, hashes (#) indicate the mass peaks corresponding to the s01 cleaved fragments sequentially attached to the M^pro^ dimer; note: the resolution is not sufficient to distinguish between the N- or C-terminal fragments (mass shifts of 617 and 593 Da, respectively); (b3) same solution as (b2) after 6 min incubation; (b4) M^pro^ and s10 solution after 1 min incubation, ‘N’ labels N-terminal fragment(s) attached (765 Da), ‘C’ labels C-terminal fragment(s) attached (560 Da); (b5) M^pro^ and s03 solution after 3 min incubation, ‘S’ labels intact substrate(s) attached, hashes (#) label attached substrate fragments, but the N- and C-terminal fragments cannot be distinguished (mass shift 644 and 566 Da, respectively); (b6) same solution as (b5) after 9 min incubation.

We then examined turnover under non-denaturing MS conditions using ammonium acetate buffer (Fig. 7a). Peptides s01, s06, s08, s10 and s11 evidenced fast turnover. The level of substrate ion depletion was >70% after 1 min and >90% after 6 min incubation. Peptides s02, s04 and s09 showed substrate ion depletion from 35 to 45% after 1 min incubation, >70% depletion after 6 min, and >90% depletion after 12 min. Peptides s03, s05 and s07 demonstrated slow turnover that was below 50% after 12 min incubation.

In the protein region of the mass spectra, complexes of the M^pro^ dimer and the cleavage products were observed after 1 min of incubation for the fast-turnover substrates s01, s06, s08, s10 and s11, and also s02, s04 and s09 (Fig. 7b1–b4). For the slow-turnover substrates s03, s05 and s07, only M^pro^ complexes with intact substrates were observed after 1 min incubation. For longer incubation times, complexes between M^pro^ and the products from these substrates emerged and increased in abundance (Fig. 7b5 and b6).

The rank order of the substrates in part depends on the MS method used, likely due to the differences in the buffers and concentrations used: *i.e.*, non-denaturing MS used ammonium acetate buffer and an M^pro^ concentration of 5 μM, which is higher than the 0.15 μM used in denaturing MS assays. Higher concentrations of both enzyme and substrate in the non-denaturing MS experiments explain the faster substrate turnover than seen with denaturing MS, especially as the concentration of catalytically active M^pro^ dimer would be higher at higher enzyme concentrations.^6,54^

Regardless of the MS method used, a clear trend is observed in the catalytic turnover of the cleavage site-derived peptides. The rank order of substrate preference under denaturing MS conditions was s01 > s11 > s06 > s02 > s10 > s07 > s09 > s05 (Fig. S2.22 and S2.23†). Under non-denaturing conditions (Fig. 7) turnover was: fast (s01, s11, s06, s10, and s08), medium (s04, s02, and s09), and slow (s05, s03, and s07). Substrates s01, s11 and s06 turned over fastest; while s07, s05 and s03 were slow as measured by both methods. This is in broad agreement with the reversed phase high performance liquid chromatography analysis of substrate turnover by SARS-CoV M^pro^, where s01 and s02 display fast turnover; s10, s11 and s06 manifest medium turnover; and the rest (s09, s08, s04, s03, s05, s07) show slow turnover.^44^ Both of our MS studies on SARS-CoV-2 M^pro^ indicate that s02 consistently displayed slower turnover than s11. Previous reports on SARS-CoV M^pro^ have shown evidence for cooperativity between subsites during substrate binding, in particular during autocleavage of the M^pro^ C-terminal site (s02), where the Phe at P2 induces formation of the S3′ subsite to accommodate the P3′ Phe residue.^55^ SARS-CoV-2 M^pro^ substrate s02 has a Phe at P2, but not at P3′ (Fig. 1a). The absence of a Phe at the P3′ position may in part explain the reduced activity of SARS-CoV-2 M^pro^ for s02 relative to s01, compared to the same pair in SARS-CoV M^pro^.^44^

The observed turnover of all 11 SARS-CoV-2 cleavage-site-derived peptides by M^pro^ is consistent with our atomistic models, where the peptides remain bound in the active site during MD simulations and where the scissile amide carbonyl remains well-positioned in the oxyanion hole (*e.g.*, HB 8 in Fig. 3) for reaction initiation. The stability of the M^pro^–peptide interactions involving the S2 and S1 subsites, as well as backbone–backbone HBs 2, 3, 10 and 11, could explain the observation using non-denaturing MS of complexes of M^pro^ with products—because of slow product dissociation. Nevertheless, we envisage that the order of substrate turnover rates is likely determined by various factors, including peptide conformations, the influence of the P2 and P1′ residues on the catalytic dyad (as highlighted by the BigDFT analysis), entropic effects, and rates of product dissociation, all of which prompt ongoing experimental and computational investigations.

## 3. *In silico* mutational analysis of substrate peptides enables peptide inhibitor design

Building on insights gained from our binding studies of SARS-CoV-2 M^pro^ and the 11 SARS-CoV-2 polypeptide substrate sequences, we designed peptides that could bind more tightly than the native substrates. We quantified the per-residue energetic contributions of these sequences to the overall binding in the M^pro^ active site and proposed substitutions that would increase affinity. We hypothesised these peptides would: (a) behave as competitive inhibitors, and (b) provide counterpoints for comparison with natural substrates, shedding light on requirements for M^pro^ binding and, perhaps, turnover.

### 3.1 *In silico* alanine scanning and predictive saturation variation scanning

We used the interactive web application BAlaS to perform Computational Alanine-Scanning mutagenesis (CAS) using BudeAlaScan^56^ and the BUDE_SM algorithm^57^ for Predictive Saturation Variation Scanning (PreSaVS).^58^ Both are built on the docking algorithm BUDE,^59^ which uses a semi-empirical free energy force-field to calculate binding energies.^60^ To identify key binding interactions of the natural substrate peptides to M^pro^, the 11 substrate:M^pro^ complexes were first subjected to CAS using BAlaS. By sequentially substituting for alanine, the energetic contribution of each substrate residue to the overall interaction energy between the singly mutated peptide and M^pro^ is calculated using:ΔΔ*G* = Δ*G*_Ala_ − Δ*G*_wt_where Δ*G*_wt_ is the interaction energy between the peptide and M^pro^, and Δ*G*_Ala_ is the interaction energy for the peptide with a single alanine mutation at a given position. The more positive the value for each residue, the greater the contribution from that substrate residue to binding. This method was used later to evaluate potential inhibitor peptides.

**Fig. 8:**
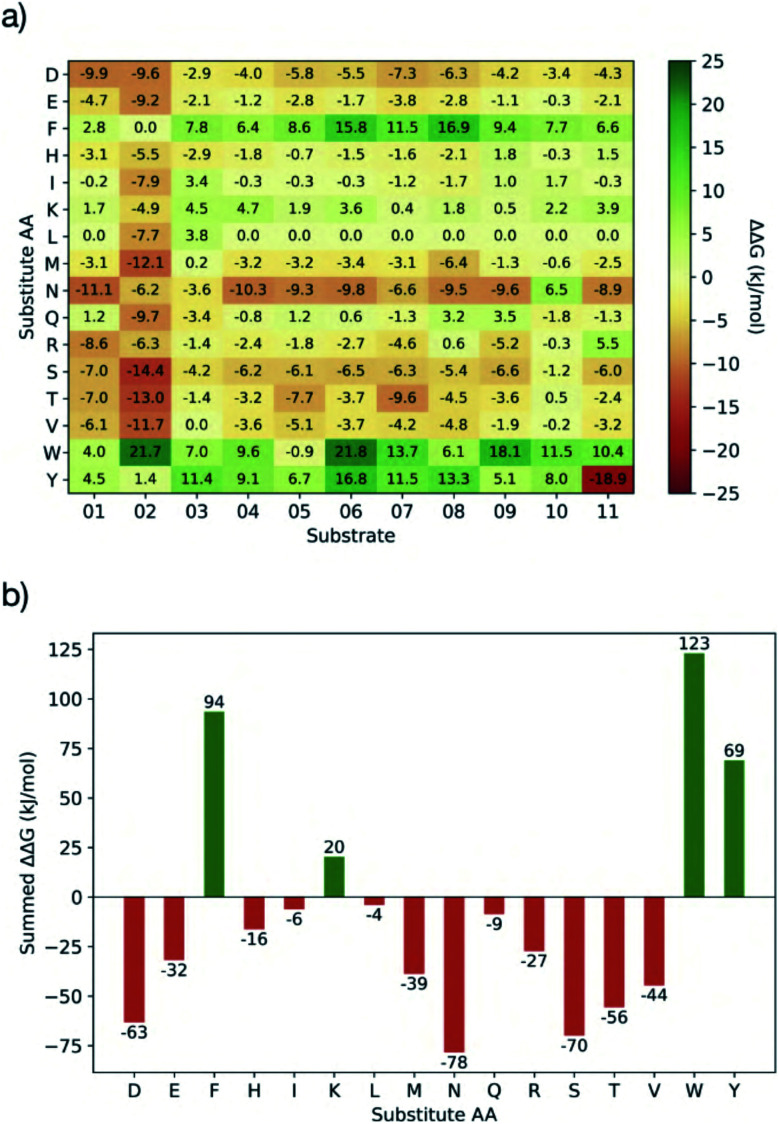
BUDE_SM PreSaVS for the P2 position. (a) Heat-map for BUDE_SM PreSaVS saturation mutagenesis at P2, showing the ΔΔ*G* = Δ*G*_wt_ − Δ*G*_mut_ value calculated for each substitution and each M^pro^ substrate. Mutations predicted to improve peptide binding have a positive ΔΔ*G* and are greener; those disfavouring binding are in red. (b) The summed ΔΔ*G* values for each residue type substituted at P2.

Having identified residues contributing most to the binding energy of the natural M^pro^ substrates, each of the sequences was subjected to PreSaVS using the BUDE_SM algorithm. This sequentially substitutes each substrate residue with a range of residues (D, E, F, H, I, K, L, M, N, Q, R, S, T, V, W and Y). BUDE_SM calculates the ΔΔ*G* = Δ*G*_wt_ − Δ*G*_mut_ for the binding interaction of each, entire, singly mutated peptide with M^pro^. Substitutions predicted to improve binding over wildtype sequences have a positive ΔΔ*G*. Fig. 8 shows an example of the BUDE_SM PreSaVS results for all the P2 substitutions for the 11 substrate peptides (normally Leu, Phe, or Val in the 11 substrates). The most positive results suggest that Phe, Trp and Tyr favour increased predicted affinity at the P2 position (Fig. 8b). However, although Tyr generally increased the predicted binding affinity (ΔΔ*G*_sum_ = 68.8 kJ mol^−1^), it was not considered for substitution at P2 due to its negative effect at this position in s11 (scoring −18.9, Fig. 8a). Candidate residues for each position, from P6 to P5′, were shortlisted similarly based on those with the best total, and the fewest unfavourable, scores.

**Fig. 9:**
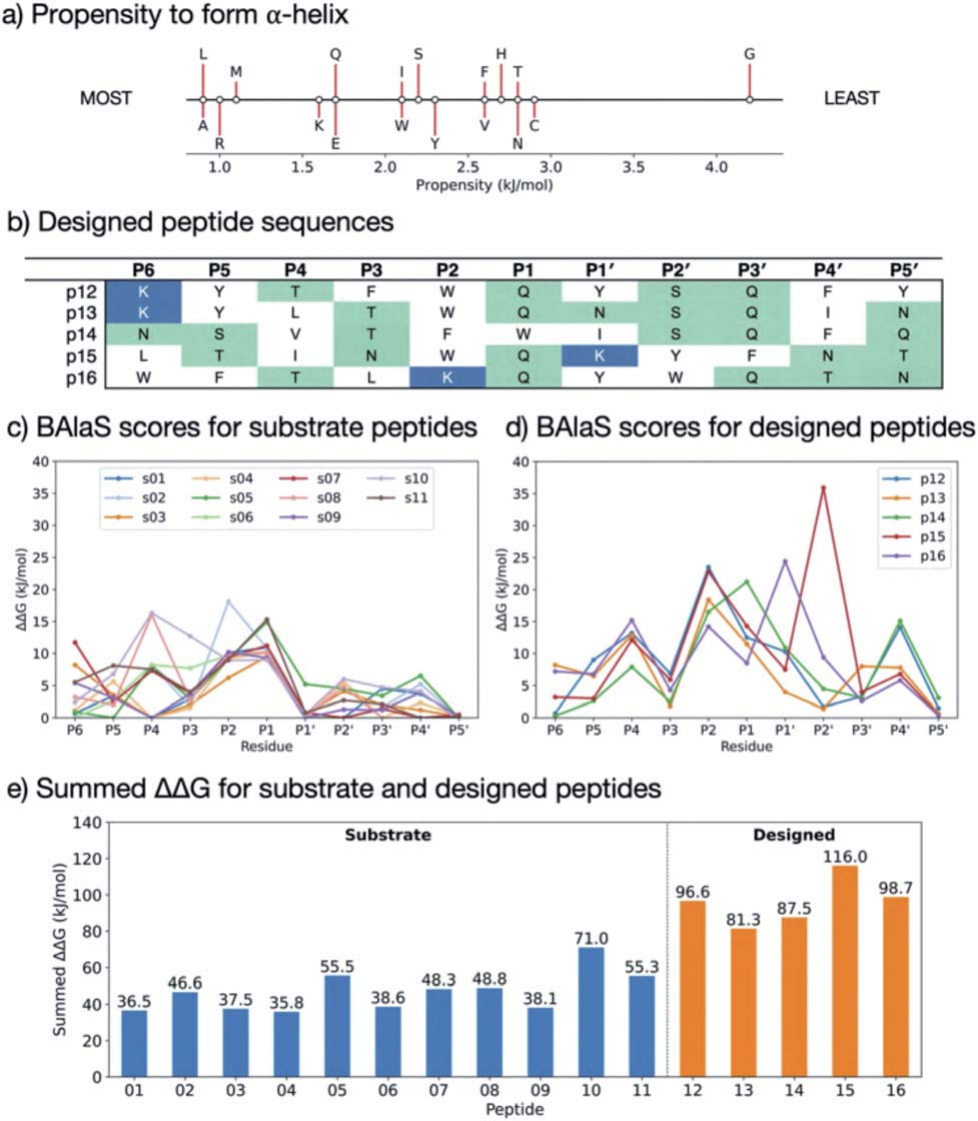
BAlaS-guided design of tight-binding peptides. (a) Propensity scale of each amino acid to form an α-helical peptide conformation. (b) Sequences of designed peptides p12–p16. Scatter plots with predicted BAlaS ΔΔ*G* = Δ*G*_Ala_ − Δ*G*_wt_ values on substitution to alanine for each residue of (c) the 11 M^pro^ natural substrates and (d) designed peptides based on these. The more positive the value, the greater the contribution made by the sidechain to the overall binding energy. (e) The BAlaS ΔΔ*G*_Sum_ comparing values between complexes of M^pro^ with substrate and designed peptides as a proxy for predicting relative binding affinity (larger score = tighter binder).

In addition to the computed ΔΔ*G* values, we considered the propensity of each residue to promote an extended conformation. All bound substrates are largely extended, so entropic penalties may be avoided if inherently extended conformations could be favoured in the designed peptide. Thus, the best β-forming (and therefore least α-forming) residues from the first triage were selected (Fig. 9a).^61^ We also considered solubility. This was achieved by limiting the number of hydrophobic residues in each designed peptide and ensuring a net positive charge (except p14, which was neutral).

### 3.2 Designed peptide sequences

Employing the criteria described above, five new peptides, p12–p16, were designed (Fig. 9b). Comparison of the computed ΔΔ*G* values for s01–s11 (Fig. 9c) and p12–p16 (Fig. 9d) reveals that substitutions at the P sites provide only occasional, moderate improvements to binding energy over the corresponding substrate P sites, with the notable exception of P2, which can accommodate Trp, Phe or Lys. These results agree with the HB analysis, which predicts that the sidechains of residues that are on the N-terminal side of the cleavage site (P sites) contribute more to binding than C-terminal, P′ sites. The most striking difference between substrates and designed peptides is in this P′ region, where the predicted binding energy contributions for the designed peptides exceed those of the substrates, an advantage that is distributed over most of the designed P′ positions.

The final step in design was to assess the relative binding affinities of the substrates and designed peptides. Hence the summed ΔΔ*G*s (Fig. 9e) provide a proxy for the binding energies (BAlaS)^62^ for the substrates and designed peptides with M^pro^. The substrate:M^pro^ complexes are stabilised by an average of 46.5 kJ mol^−1^, whereas the designed-peptide:M^pro^ complexes are predicted to have, in some cases, double the interaction stability of the substrates, with an average of 96.0 kJ mol^−1^. The full analysis is in the ESI file SI_BAlaS_BUDE_SM_12-04-2021.xlsx.†

### 3.3 Synthesis and analysis of designed peptides

**Fig. 10:**
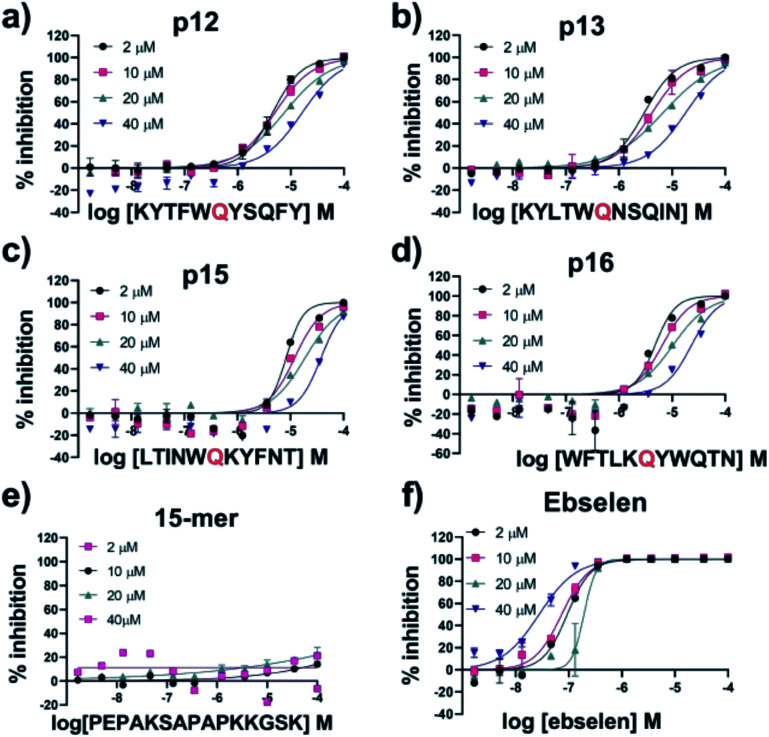
IC_50_ of designed peptides against M^pro^ with varied substrate concentrations. IC_50_s for (a) p12, (b) p13, (c) p15, (d) p16, (e) 15-mer control peptide and (f) ebselen with 2 μM, 10 μM, 20 μM and 40 μM of substrate peptide s01. IC_50_ values were calculated from technical duplicates (Table S3.2†). See Experimental Section S1.8† for assay details.

To test the designed sequences, p12, p13, p15 and p16 were synthesised with a carboxyl-amide C-terminus by solid phase synthesis. Their M^pro^ inhibitory activity was determined by dose–response analysis (Table 1) using SPE MS, monitoring both substrate s01 (1191.68 Da) depletion and N-terminally cleaved product (617 Da) formation. Ebselen which reacts multiple times with M^pro^^63^ was used as a standard (IC_50_ = 0.14 ± 0.04 μM; Fig. 10).

**Table 1:** Designed peptides inhibit M^pro^ in a dose-dependent manner. The assay conditions were 0.15 μM M^pro^, 2 μM s01 in 20 mM HEPES, pH 7.5, and 50 mM NaCl

Peptide	IC_50_/μM	Hill slope
p12	5.36 ± 2.17	1.25 ± 0.06
p13	3.11 ± 1.80	0.94 ± 0.09
p15	5.31 ± 1.08	1.17 ± 0.16
p16	3.76 ± 0.51	1.19 ± 0.16

All four designed peptides manifested similar potency with IC_50_ values ranging from 3.11 μM to 5.36 μM (Table 1 and Fig. S3.1†). Strikingly, despite the presence of Gln at P1 in all the designed peptides assayed, no evidence for hydrolysis was observed by SPE MS. This observation was supported by LCMS of the peptides incubated overnight with M^pro^ (Fig. S3.2†). We probed the inhibition mode of the designed peptides by monitoring changes in IC_50_ while varying the substrate concentration (2 μM, 10 μM, 20 μM and 40 μM TSAVLQ↓SGFRK-NH_2_ s01; *K*_m_ ∼ 14.4 μM).^63^ The results indicated a linear dependency between substrate concentration and IC_50_ values (Fig. 10a–d). This was not observed with a control 15-mer peptide or ebselen (Fig. 10e and f). Analysis of the data by the procedure of Wei *et al.*^64^ implies competitive inhibition (Fig. S3.3 and Tables S3.1, S3.2†). By contrast, the same analysis for ebselen did not support competitive inhibition, consistent with MS studies showing it has a complex mode of inhibition.^63^

Three of the synthesised peptides—p12, p13, and p15—have a Trp at P2 (Fig. 9b) while the other, p16, has a Lys at P2. The 11 M^pro^ substrates all have hydrophobic residues (Leu, Val or Phe) at P2 (Fig. 1a). To investigate if the nature of the hydrophobic P2 residue, or the hydrophilic nature of the Gln at P1, alters the interaction of the peptide and hence its reactivity at the active site, we synthesised p13-WP2L, s01-LP2W, and s01-QP1W. There was no evidence for cleavage of p13-WP2L or s01-QP1W. However, s01-LP2W underwent partial cleavage (12.6 ± 4.5)% after overnight incubation. These results suggest that the presence of a Trp at P2 hinders catalytically productive binding, at least with these peptides, and that other residues (including the P1′ and P2′ residues) play roles in orienting the substrates for cleavage (*vide infra*).

**Fig. 11:**
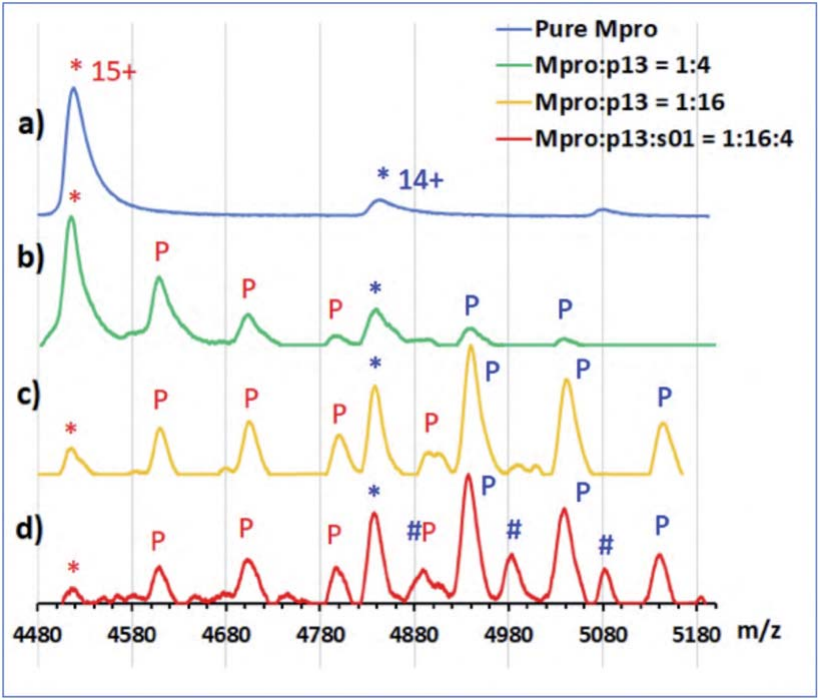
Non-denaturing MS analysis of designed peptides binding to the M^pro^ dimer. Inhibitor binding from non-denaturing MS showing normalized intensity in the *m*/*z* region around the 14+ and 15+ charge states of the M^pro^ dimer. (*) indicates unbound M^pro^ dimer. (a) 5 μM M^pro^ solution; (b) 4-fold excess of p13 relative to the M^pro^ dimer; ‘P’ indicates sequential binding of p13 peptides to M^pro^ in the 15+ charge state (red) and 14+ state (blue); (c) 16-fold excess of p13; (d) 16-fold excess of p13 and 4-fold excess of s01; hash (#) indicates sequential binding of s01-cleavage products (note: the resolution is not sufficient to distinguish between the N- and C-terminal fragments; some non-specific binding of p13 is also observed in (c) and (d) due to the high concentration of the peptide).

We then used non-denaturing protein MS to study enzyme–substrate/product/inhibitor complexes simultaneously with turnover. Complexes between M^pro^ dimer and p12 and p13 were observed, together with the uncomplexed M^pro^ dimer in the protein region of the mass spectra. No binding was observed for p15 and p16, due to relatively high noise in that *m*/*z* region. None of the designed peptides were cleaved by M^pro^, as recorded in the peptide region. As a control, s01 was added to the protein/inhibitor mixtures; for all the inhibitors, turnover of s01 was observed after 3 min incubation. Depletion of s01 was 95%, 91%, 70% and 78% in the presence of p12, p13, p15 and p16, respectively, with an 8-fold excess of inhibitor over M^pro^, *versus* >98% depletion for the M^pro^/s01 mixture without the inhibitor. In the protein region of the mass spectra, complexes between M^pro^ dimers and the s01-cleavage products were observed in the presence of p13, but the abundance of these complexes was lower than the abundance of M^pro^/p13 complexes (Fig. 11). These results validate the above-described evidence that the peptide inhibitors both bind and competitively inhibit M^pro^.

### 3.4 Understanding the basis of SARS-CoV-2 M^pro^ inhibition by the designed peptides

#### 3.4.1 Modelling of the designed peptides

**Fig. 12:**
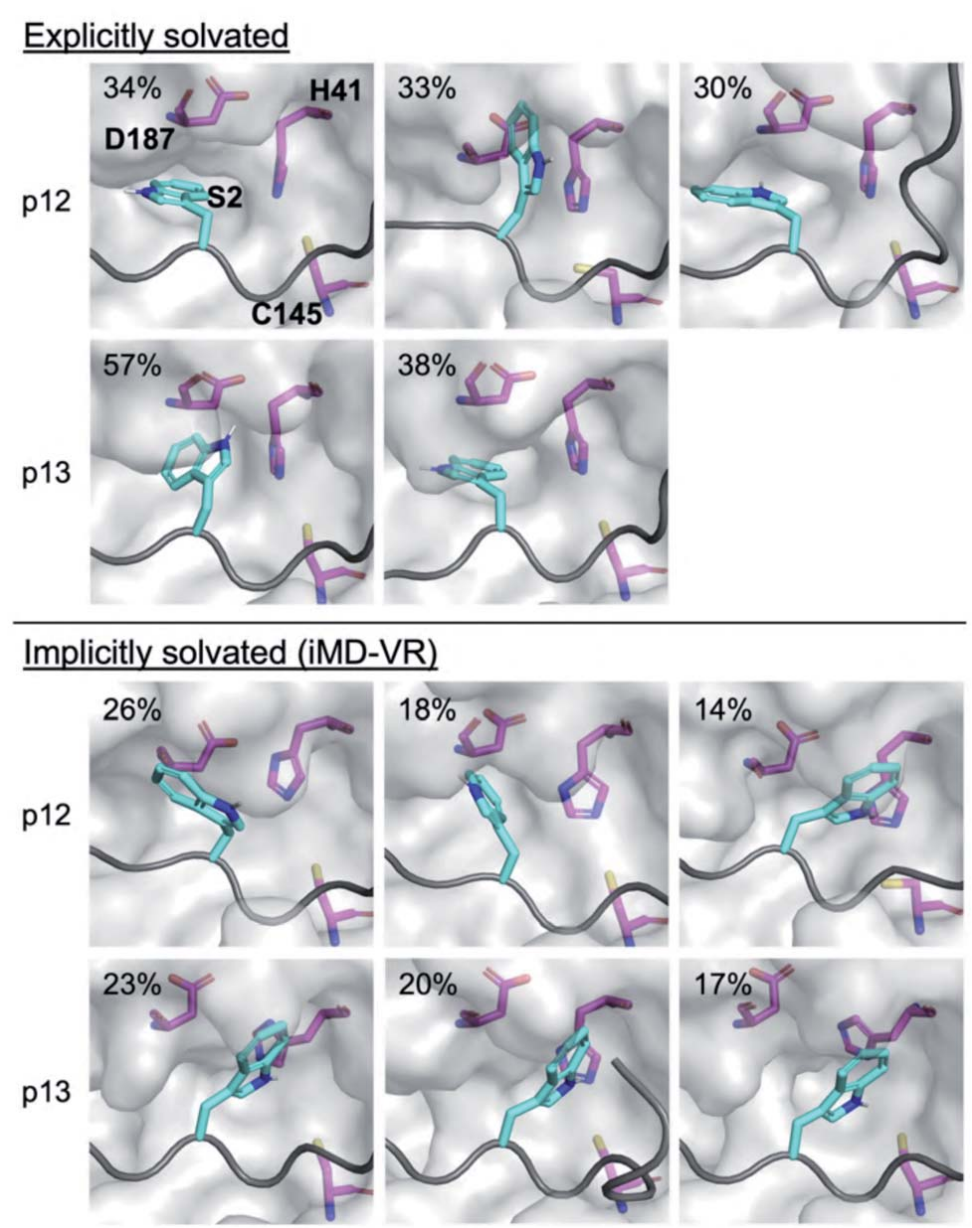
Binding of P2 Trp in the designed peptides. Conformations adopted by the P2 Trp sidechain (cyan sticks; non-polar hydrogens omitted for clarity) in p12 and p13 (grey ribbon) observed during explicit and implicit solvent MD simulations, showing representative structures obtained by RMSD clustering. His-41, Cys-145 and Asp-187 are shown in magenta. See Fig. S3.13† for cluster populations formed during MD. For each peptide, conformations are displayed in decreasing order of occurrence (above 10%).

Modelling of p12 and p13 shows that both bind stably at the active site during MD simulation (Fig. S3.4–S3.8†). Like the natural substrates, key HBs form with Glu-166, Thr-26, Thr-24, and the oxyanion hole-contributing Cys-145 (Fig. S3.9–S3.11†). However, HBs involving the P1-Gln sidechain of p12 and p13 showed greater variability. The favourability of the P2 Trp mutation, as predicted by the BAlaS scores, prompted us to investigate its binding. In line with the plasticity observed at S2, a variety of conformations are observed during MD simulations at this position, showing varying degrees of immersion in S2 (Fig. 12 and S3.12, S3.13†). Similar results were obtained using iMD-VR (Fig. 12).

**Fig. 13:**
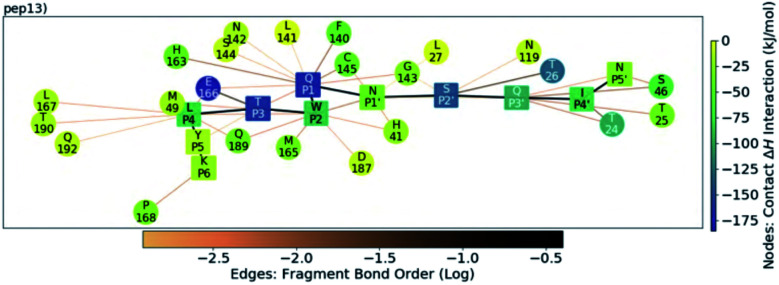
QM contact interaction graph for p13 and M^pro^. Interactions are computed using ensemble-averaged results of MD snapshots with the BigDFT code.^50^

Analysis of the conformations of the most populated cluster from MD using Arpeggio-generated hydrophilicity maps (Fig. S3.14†) reveals that the P2 Trp is more deeply buried within S2 than the native P2 residues in the natural substrates, forming more than double the number of hydrophobic contacts in the cases of p12 and p13. Some conformations involve the indole ring π–π-stacking, or hydrogen-bonding *via* its indole N–H, with the catalytic His-41 sidechain, forming an extended HB network (Fig. 12). It is possible that these interactions may hamper the ability of His-41 to deprotonate Cys-145 at the start of peptide hydrolysis, which could be tested using QM/MM calculations. Interestingly, DFT-based interaction analysis reveals that one of the slowest turnover substrates, s05 (Fig. 5), and inhibitor p13 (Fig. 13), share similar short-range interaction networks.

#### 3.4.2 Comparative peptide docking

To investigate the ability of the M^pro^ subsites to recognise residues in the designed sequences, AutoDock CrankPep (ADCP) was used (Table S3.3†).^65^ A trial was performed by redocking s01 into the H41A SARS-CoV M^pro^ structure originally complexed with s01 (PDB entry 2q6g).^37^ ADCP successfully positioned the peptide mostly correctly in its top solution, with the C_α_ positions from P5 to P1′ deviating by less than 1 Å (Table S3.4 and Fig. S3.15†). Deviations increased up to 16 Å at P5′ as the peptide coiled up in the P′ positions, but this is deemed acceptable since the S′ subsites are less well defined, as discussed earlier.

Following the promising redocking results with ADCP, s01–s11, p12, p13, p15, and p16 were docked with an M^pro^ structure originally complexed with the N3 inhibitor (PDB entry 7bqy; 1.7 Å resolution).^2^ Substrate-docked structures were found to have the P4 and P2 residues correctly positioned in their corresponding S4 and S2 pockets (Table S3.5†). From P1 to P5′ the poses were more variable, with some peptide backbones turning through S1 rather than continuing an extended conformation, likely due to the less well-defined S′ subsites (Fig. S3.16†). For the designed peptides, by contrast, docking appeared less successful (except p16), with none of the top 10 solutions positioning the peptide in the manner observed in our MD simulations (Fig. S3.17†). The S2 pocket in 7bqy binds the Leu sidechain of N3 and is probably too shallow to accommodate the larger Trp sidechain, given the assumption of a rigid receptor in ADCP docking. Hence, the four designed peptides were also docked to the C145A M^pro^ structure in complex with the s02 cleaved product (PDB entry 7joy; 2 Å resolution),^66^ which has a deeper S2 pocket that binds the P2 Phe sidechain in s02. Interestingly, for both p12 and p16, the top docked solution matched our design more closely (Table S3.6 and Fig. S3.18†). Docking of p13 and p15 was challenging, possibly due to the difficulty of recognising a larger Leu (p13) or Ile (p15) residue in the S4 pocket, which originally accommodated a Val sidechain. This highlights the ability of the M^pro^ active site to adapt when binding to different substrates or inhibitors.

### 3.5 Summary – designed peptides

We used *in silico* Predictive Saturation Variation Scanning to design peptides that were shown *in vitro* to inhibit M^pro^ competitively. Structures of p12 and p13 generated by both iMD-VR docking and comparative modelling behaved similarly, in terms of HB formation and peptide backbone RMSD and RMSF, when performing MD. These studies highlight how the S2 subsite can adapt its size and interaction network *via* induced fit to accommodate different substrate or inhibitor P2 residues.

Notably, while these models suggested similarly stable binding modes as seen with the natural substrates, turnover of the inhibitor peptides by M^pro^ was not detected. This may relate to the more favourable predicted binding affinity of the designed peptide–M^pro^ complexes, both in terms of higher overall interaction energies, and greater contribution of the P′ residues than in the natural substrates. Our MD simulations suggest it is also possible that the larger P2 residue prevents the catalytically vital His-41 from adopting a reactive conformation (Fig. 12).

## 4. M^pro^–ligand interaction analysis

Having elucidated how M^pro^ recognises its substrates and our designed peptide inhibitors, we hypothesised that this might be reflected in the extensive small-molecule inhibitor work on M^pro^ and could, in turn, be exploited for the design of novel small-molecule inhibitors and peptidomimetics. We explored whether ligands sharing the same contacts as the natural substrates could lead to better inhibitory activity. We analysed all 91 X-ray structures of small molecule fragments complexed with M^pro^ obtained by high-throughput crystallographic screening at Diamond's XChem facility,^35^ as well as the dataset of 798 designed inhibitors and 245 crystal structures obtained from the COVID Moonshot project.^36^ We analysed them by investigating their protein–ligand interaction patterns.

### 4.1 Interaction analysis of XChem fragments

**Fig. 14:**
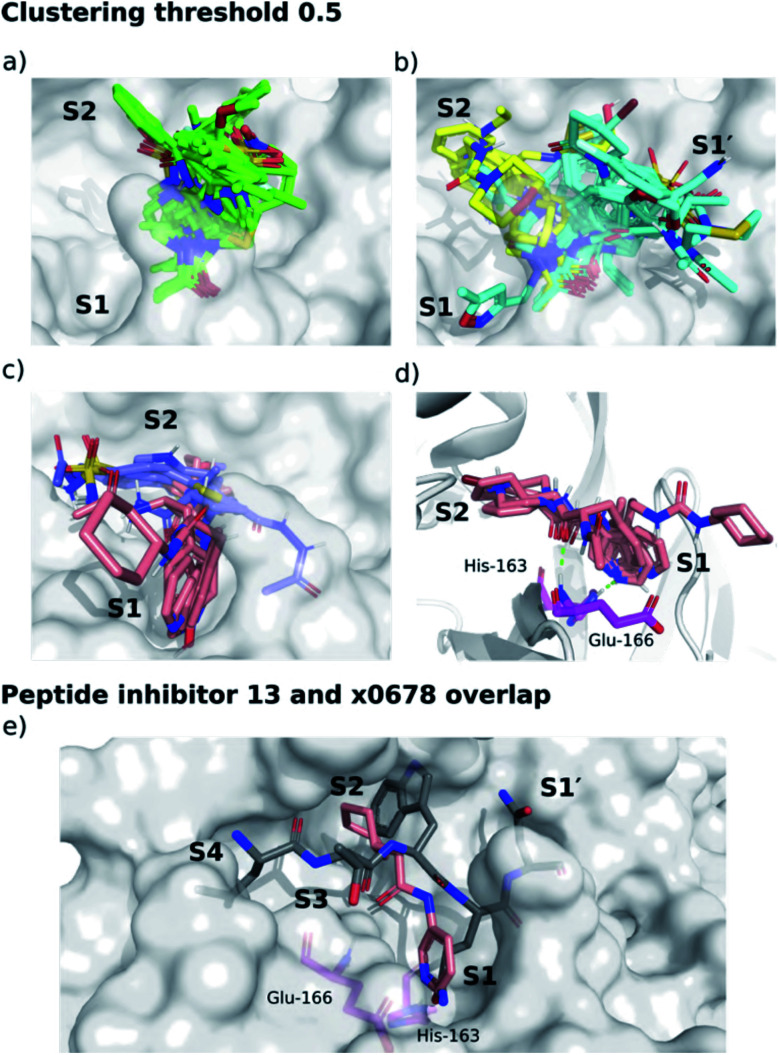
Clustering of XChem active site-binding fragments. Surface of the x0830-bound M^pro^ structure (white surface) and the top 5 most populated fragment clusters using a clustering threshold of 0.5. (a) Cluster 1 fragments tend to occupy S1′ (green); (b) clusters 2 (cyan) and 3 (yellow) tend to span S1′ and S2; (c) clusters 4 (lilac) and 5 (pink) tend to occupy S2 and S1. (d) Close-up of cluster 5. Green dotted lines indicate the two key HBs between the fragment carbonyl oxygen and the backbone nitrogen of Glu-166 (HB 3, Fig. 3), and between the His-163 Nε and the heterocyclic nitrogen of the fragment (HB 6, Fig. 3). (e) Overlay of the P4–P1′-truncated structure of peptide inhibitor p13 (grey) from an MD snapshot and cluster 5 binder x0678 (pink), with the x0678 co-crystal structure (white surface).

We separated fragments into non-active-site binders (25 fragments) and active-site binding/likely-substrate competing molecules (66 fragments; Fig. S4.1†). A fingerprint bit-vector was constructed for every active-site binding fragment, with each bit denoting the presence or absence of a given interaction with M^pro^ residues, and used for clustering fragments by their interaction fingerprint Tanimoto similarity,^67^ with 1 corresponding to identical contacts, and 0 to no shared contacts (Fig. 14, S4.2–S4.5 and Table S4.1†). All the fragments and ligands in clusters 1 and 2 (except x0397, x0978 and x0981) are covalently bound to Cys-145. As a result, a highly conserved binding mode is observed for the carbonyl-containing covalent warheads (*e.g.*, chloroacetamides), where the carbonyl oxygen binds into the oxyanion hole between residues Gly-143 and Cys-145, mimicking substrate HBs 8 and 9 (Fig. 3). Cluster 5 stands out as the only major cluster with fragments that bind deeply into S1, one of the main conserved contacts identified in all substrates. Cluster 5 shows a distinct binding motif primarily driven by: (i) hydrogen bonding between a carbonyl oxygen on the fragment and the Glu-166 backbone NH-group; and (ii) a strong polar interaction between His-163 and the fragment. Notably, the protonation of the imidazole of His-163 appears to depend on the fragment.

**Fig. 15:**
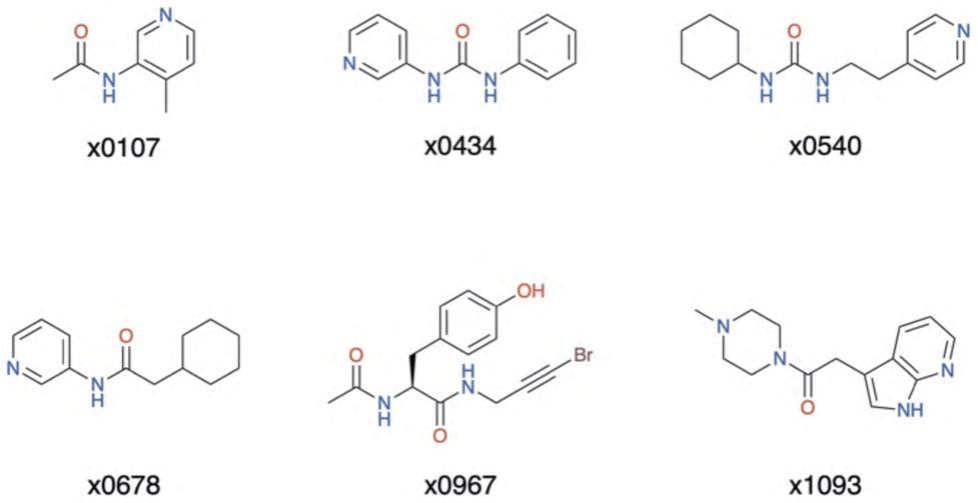
Structures of the cluster 5 XChem compounds. Note the prevalence of nitrogen-containing heterocycles, and the phenol-containing derivative x0967.

Overall, the primary functionality that facilitates interaction with His-163 is the nitrogen-containing heterocycle present in almost all ligands in cluster 5 (Fig. 15); the exception is x0967, which forms the His-163 HB *via* its phenol oxygen. Such heterocycles are well suited to replace the substrate P1 Gln sidechain by mimicking its HB donor/acceptor abilities. In addition, most cluster 5 binders also extend into the hydrophobic S2 pocket, although there is no clear preference in functional group at S2. This agrees with our plasticity analysis, which shows that S2 can accommodate a large variety of functional groups (Fig. S4.4†). As seen in the overlap of peptide inhibitor p13 and cluster 5 representative x0678 (Fig. 14e), the binding modes of both inhibitors in the S1 and S2 subsites are very similar, with both forming HBs to His-163 (HB-6) and Glu-166 (HBs 2 & 3) and binding deep in the S2 pocket. In addition, all cluster 5 ligands (Fig. 15) contain an amide or urea linker between the P1 and P2 binding groups making them interesting building blocks for the development of peptidomimetics.

The interactions between the fragments, substrates, and peptide inhibitors with M^pro−^ were analysed by employing linear scaling DFT. Using short-range (*E*_cont_) DFT interactions with M^pro^ as a “descriptor” for clustering, a cluster containing both the substrates and the cluster 5 compounds (x0107, x0434, x0540, x0678, x0967 and x1093) was identified (Fig. S4.6†). This cluster also included compounds x0426, x0946, x0195, x0995, x0104, x0874, x1077, x0161 and x0397. This agnostic analysis, based on quantum mechanical descriptors, provides further confirmation and a powerful alternative to evaluate compounds of differing sizes in biomolecular complexes.

To test whether cluster 5 inhibitors are promising building blocks for optimization, we identified all assayed and crystallized cluster 5 binders in the COVID Moonshot project database^36^ as of 11th Jan 2021 and analysed them using Arpeggio. Compounds were deemed cluster 5 inhibitors if they shared at least 70% of the contacts identified in fragment cluster 5. We observed that cluster 5 inhibitors have a significantly higher proportion of “strong” binders, classified as IC_50_ < 99 μM (85% of cluster 5 compounds), unlike the rest of the Moonshot project database (67%). Closer analysis can be found in Section S4.2 and Fig. S4.7.† In summary, based on Arpeggio and BigDFT contact analysis and reported assay data, cluster 5 binders are promising building blocks for substrate-competing inhibitor design.

### 4.2 Covalent docking of COVID Moonshot compounds

**Fig. 16:**
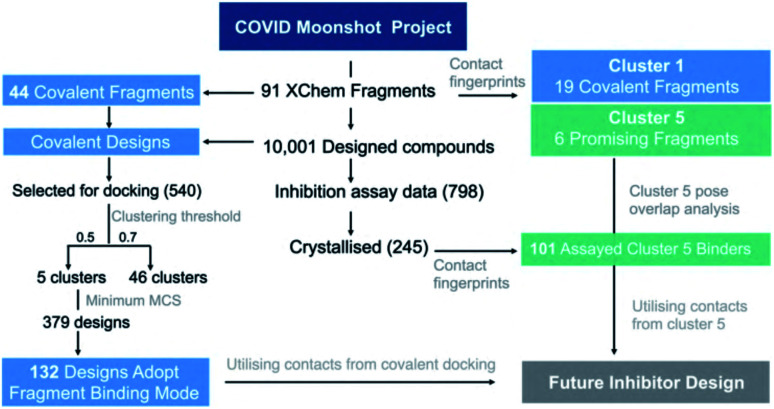
Analysis of fragment and designed compounds from the Moonshot project. Workflow used to identify promising fragments and guide novel designs.

To accommodate induced fit and create high quality poses of covalent inhibitors for future optimisation, we selected 540 covalently-reacting compounds from 10 001 Moonshot-designed compounds and docked them using AutoDock4 ^68^ into the M^pro^ structure of the corresponding covalent “inspiration” fragments.^36^ We generated an interaction Tanimoto distance matrix as described earlier, and analysed the ability of the procedure to recapitulate the binding mode of the parent fragment. The normalized shape and pharmacophoric overlap (SuCOS^69^) of the lowest energy pose of the highest populated cluster for each Moonshot compound was compared with the inspiration covalent XChem fragment (Fig. S4.8†). When controlling for the smallest maximum common substructure (MCS) that encompasses the covalent warhead and one additional atom in the compound, 379 designs remain, 132 (34.8%) of which adopted the binding mode of the inspiration fragment. Given the high similarity between the fragments and docked designs, it is likely that these binding modes are more representative of the actual binding mode. A summary of the workflow is shown in Fig. 16.

In summary, our covalent docking method is more likely to identify the correct binding mode when substantial overlap exists between the inspiration fragment and designed compound beyond the covalent warhead (Section S4.3 and Fig. S4.8, S4.9†). This generated 132 high quality docked poses which serve as inspiration for future inhibitor design and were used in our proposals for compound derivatisation in Section 4.3. All poses of the 540 docking runs are available at https://github.com/gmm/SARS-CoV-2-Modelling.

### 4.3 Implications for future inhibitor design

We compared the interactions of the cluster 5 binders with those in the substrates, peptide inhibitors, and XChem fragments. Interestingly, unlike the peptides, almost none of the cluster 5 binders interact with the oxyanion hole. The only cluster 5 compounds where this contact is made are a series of covalent inhibitors, none of which showed promising potency (Fig. S4.11†). An exhaustive search of Moonshot structures showed that at the time of the analysis, no non-covalent inhibitor has ever been tested that includes both the typical cluster 5 binding mode while also being able to interact with the oxyanion hole.

We compared the structures of the top 10 compounds in cluster 5 (part of the dataset analysed in Section 4.1) to the docked structures of covalent Moonshot designs (Section 4.2). Two compounds—FOC-CAS-e3a94da8-1 and MIH-UNI-e573136b-3—were selected based on their high normalized SuCOS overlap with their inspiration fragments, strongly suggesting that their docked binding modes reflect the actual poses.^70^ Both compounds bind into the oxyanion hole as well as into S1 and S2, providing a clear opportunity for extension of the cluster 5 binders (Fig. S4.12†).

**Fig. 17:**
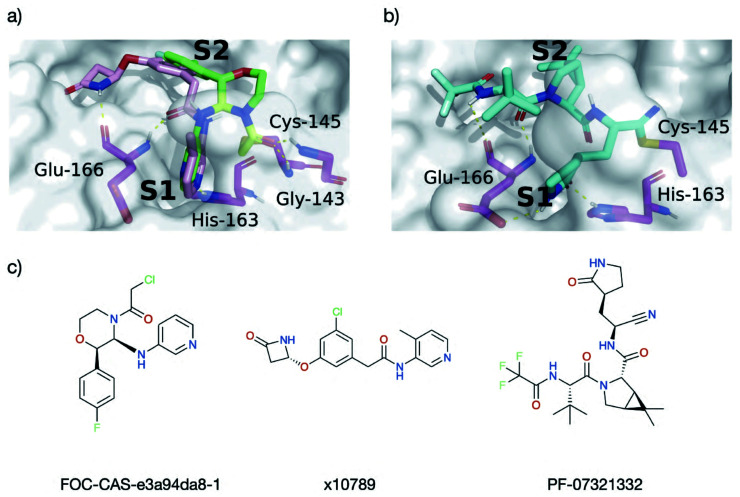
Docking informs novel inhibitor design. HBs between M^pro^ (magenta) and the ligands are shown as dotted yellow lines. (a) Overlay of the docked pose of FOC-CAS-e3a94da8-1 (green and greenish-yellow) with the crystal structure of x10789 (pink) on the M^pro^ surface (PDB entry 5RER; 1.88 Å resolution).^35^ Derivatisation of x10789 into the oxyanion hole could be achieved by attaching a methylene amide group present in x0830 (highlighted greenish-yellow). (b) Docked pose of Pfizer's Phase I covalent inhibitor PF-07321332, covalently docked into M^pro^ (PDB entry 6XHM; 1.41 Å resolution).^71^ PF-07321332 (cyan) is covalently attached to Cys-145. The docked PF-07321332 adopts the same major contacts as the ‘combination’ of x10789 and x0830, namely the double HB to the backbone of Glu-166, the HB to His-163 in the S1 subsite, and a series of hydrophobic interactions in the S2 subsite. (c) Structures of Moonshot designed compound FOC-CAS-e3a94da8-1, crystallographic fragment x10789, and inhibitor PF-07321332.

Most cluster 5 binders place the aromatic heterocycle into the S1 site and the carbonyl oxygen of the amide linker bonds to Glu-166 (Fig. 17). The position of this amide nitrogen overlays perfectly with the ring amine present in the docked compound FOC-CAS-e3a94da8-1. Thus, extension of cluster 5 binders into the oxyanion hole could be achieved by adding a substituent at the amide nitrogen. A promising candidate for extension is x10789, which makes a HB with the backbone oxygen of Glu-166 (Fig. 17a) and mimics the non-prime side binding mode of peptide inhibitor p13 (Fig. 14e), even binding into the S4 site *via* its β-lactam ring (Fig. 17a). Additional expansion into the oxyanion hole and S1′ through the amide linker could yield a powerful peptidomimetic inhibitor, combining protein interactions observed for the substrates, peptide inhibitors and small molecule fragments.

When comparing interactions exhibited by cluster 5 binders (Glu-166, His-163) or covalent fragments (Gly-143, Cys-145) with the contacts present in the docked structure of the recently published Phase 1 clinical trial candidate PF-07321332 by Pfizer (Fig. 17b),^13,14^ a nearly identical interaction pattern to the cluster 5 binding motif is observed. However, note that for reacted PF-07321332, AutoDock4 was unable to place the negatively charged azanide nitrogen in the oxyanion hole, which is the expected position given its similarity to related warheads previously docked (Fig. S4.9†).

## 5. Conclusions

**Fig. 18:**
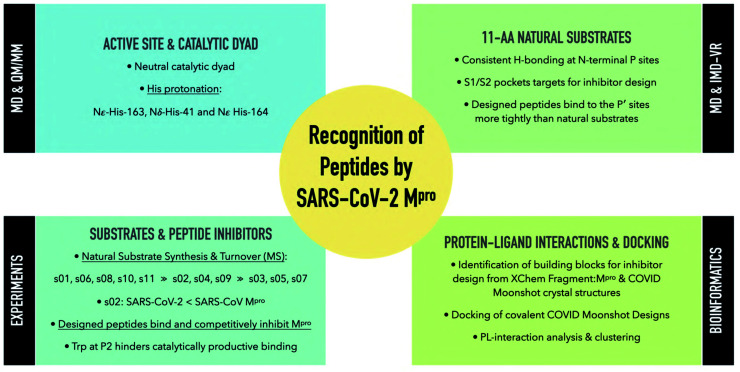
Summary of the research and results obtained in this work.

A wealth of crystal structures of SARS-CoV-2 M^pro^ is available, including hundreds with ligands. There is thus the question of how best to use this static information to help develop M^pro^ inhibitors optimised in terms of efficacy and safety for COVID-19 treatment. The dimeric nature of M^pro^, coupled with its multiple substrates, makes it challenging to understand the structural and dynamic features underpinning selectivity and catalysis, as is the case with many other proteases. Such an understanding is, of course, not essential to develop medicines, as shown by work with other viral proteases. However, it may help improve the quality of such medicines and the efficiency with which they are developed. It will also lay the foundation for tackling anti-COVID-19 drug resistance—a challenge we will likely encounter as experience with the HIV global pandemic implies. The scale of global efforts on M^pro^ makes this system an excellent model for collaborative efforts linking experimental biophysics, modelling, and drug development (Fig. 18).

The results of our combined computational studies, employing classical molecular mechanics and quantum mechanical techniques, ranging from automated docking and MD simulations to linear-scaling DFT, QM/MM, and iMD-VR, provide consistent insights into key binding and mechanistic questions. One such question concerns the protonation state of the ‘catalytic’ His-41/Cys-145 dyad, an important consideration in the rates of reaction of covalently linked M^pro^ inhibitors which ultimately relates to their selectivity and potency. Our results indicate that a neutral catalytic dyad is thermodynamically preferred in M^pro^ complexed with an unreacted substrate, justifying the neutral state for MD simulations. A more reactive thiolate anion may be deleterious to the virus, as it will be susceptible to reaction with electrophiles. Importantly, analysis of the active site suggests that the precise mechanism of proton transfer in the His-41/Cys-145 dyad involves dynamic interactions with other residues, including His-163, His-164, Asp-187, and a water hydrogen bonded to the latter two residues and His-41. Proton transfer may be considered a relatively simple part of the overall catalytic cycle—these results thus highlight how M^pro^ catalysis is likely a property of (at least) the entire active site region, with a future challenge being to understand motions during substrate binding, covalent reaction, and product release.

The models we have developed of M^pro^ in complex with its 11 natural substrates provided a basis for analysis of key interactions involved in substrate recognition and for comparison with (potential) inhibitor binding modes. Notably, the P′ (C-terminal) side of substrates appears to be much less tightly bound than the P (N-terminal) side, where there is remarkable consistency in the hydrogen bonding patterns across the substrates. This difference may in part reflect the need for the P′ side to leave (at least from the immediate active site region) after acyl–enzyme complex formation and prior to acyl–enzyme hydrolysis. The tighter binding of the N-terminal P-side residues suggests these are likely more important in substrate recognition by M^pro^. This is also reflected in potent inhibitors, such as N3 and peptidomimetic ketoamides,^2,4^ which predominantly bind in these non-prime S subsites. The development of S-site-binding inhibitors may also reflect the nature of the substrates used in screens leading to them, which typically comprise an S-site binding peptide with a C-terminal group enabling fluorescence-based measurement. Our results imply that there is considerable scope for developing inhibitors exploiting the S′ subsites, or both S and S′ subsites, though relatively more effort may be required to obtain tight binders compared to targeting the S subsites.

Consistent with prior studies, our work highlights the critical role of the completely conserved P1 Gln residue in productive substrate binding and analogously in inhibitor binding. However, the nature of the P2/S2 interaction is also important in catalysis. In the natural substrates (Fig. 1), the P2 position is Leu in 9 of the 11 substrates, Phe in s02 (which displays medium turnover efficiency), and Val in s03 (which is a poor substrate). Our results show that the S2 subsite plays a critical role in recognition and inhibition. S2 is highly plastic (Fig. 6 and S4.4†) and can accommodate a range of different sidechains, including larger groups, though not necessarily in a productive manner. The observation that substrates with a P2 Leu vary in efficiency reveals that interactions beyond those involving P1 and P2 are important, reinforcing the notion that (likely dynamic) interactions beyond the immediate active site are important in determining selectivity both in terms of binding and rates of reaction of enzyme–substrate complexes.

Notably, the results of computational alanine scanning mutagenesis followed by design, aimed at identifying peptides that would bind more tightly than the natural substrates, led to the finding that substitution of a Trp at P2 ablates hydrolysis creating an inhibitor. The observations with peptide inhibitors of M^pro^ have precedence in studies with other nucleophilic proteases, including the serine protease elastase, showing that substrate substitutions away from the scissile P1/P′ residues can cause inhibition.^72,73^ There is thus scope for the extensive development of tight binding peptidic and peptidomimetic M^pro^ inhibitors for use in inhibition and mechanistic/biophysical studies, with the Trp at P2 of the peptide inhibitors being a good point for SAR exploration, potentially by (i) replacement of the indole hydrogen with suitable alkyl or aryl substituents; (ii) introduction of substituents with different stereoelectronic properties at C-2 or C-5 of the indole ring; or (iii) cyclization by the insertion of a methylene group linking position 2 of the indole ring to the α-nitrogen of Trp itself.^74^

Finally, the combined analysis of interactions involved in substrate binding and extensive structural information on inhibitor/fragment binding to M^pro^ enabled us to identify a cluster of inhibitors whose interactions relate to those conserved in substrate binding (*e.g.*, involving the Glu-166 backbone, His-163 sidechain, and/or the oxyanion hole formed by the Cys-145 and Gly-143 backbones). Building out from these ‘privileged’ interactions (Fig. 17) might be a useful path for inhibitor discovery. Indeed, an M^pro^ inhibitor now in clinical trials^13,14^ exploits the same ‘privileged’ interactions that we identified. We hope the methods and results that have emerged from our collaborative efforts will help accelerate the development of drugs for treatment of viral infections, and particularly COVID-19.

## 6. Methods

A detailed description of the experimental and computational methods employed in this work is provided in the ESI.†

## Data availability

Structures, input files, underlying data, and source code are publicly available on GitHub at https://github.com/gmm/SARS-CoV-2-Modelling.

## Author contributions

G. M. M. generated the initial 11 natural substrate-SARS-CoV-2 M^pro^ models. R. M. T. carried out QM/MM studies with subsequent contributions from K. Ś. and V. Mo.; H. T. H. C. carried out classical MD simulations and ADCP peptide docking. R. K. W. and H. M. D. performed iMD-VR simulations. M. A. M. performed protein–substrate, protein–peptide inhibitor and protein–ligand contact analysis, fragment clustering, COVID Moonshot covalent docking and future inhibitor suggestions. M. A. M. and L. G. performed bioinformatic study of fragment binding to M^pro^. L. G. performed linear-scaling QM-DFT calculations, and W. D., T. N. and T. J. W. contributed with its setup and analysis. D. K. S. carried out mutagenesis analysis and designed novel peptides (using software devised by R. B. S.). E. S., P. L., C. S. D., C. D. O. and M. A. W. carried out M^pro^ production and purification. T. R. M. and T. J. synthesised and purified natural and designed peptides. T. R. M. also performed kinetic analyses. V. M. undertook non-denaturing MS analyses. V. Mo. and A. L. contributed to discussions. C. J. S., A. J. M., D. K. S., F. D. and G. M. M. conceptualised and supervised the study. H. T. H. C., R. K. W., M. A. M., T. R. M., R. M. T., V. M., C. J. S., D. K. S., L. G., A. J. M., F. D. and G. M. M. wrote the manuscript.

## Conflicts of interest

There are no conflicts to declare.

## Supplementary Material

SC-012-D1SC03628A-s001
